# One case of arthrogryposis-renal dysfunction-cholestasis (ARC) syndrome featuring an incomplete and mild phenotype

**DOI:** 10.1186/s12882-022-02851-2

**Published:** 2022-06-27

**Authors:** Lianhu Yu, Dan Li, Ting Zhang, Yongmei Xiao, Yizhong Wang, Ting Ge

**Affiliations:** Department of Gastroenterology, Hepatology and Nutrition, Shanghai Children’s Hospital, School of Medicine, Shanghai Jiao Tong University, Shanghai, 200062 China

**Keywords:** Autosomal recessive disorder, Child, Compound heterozygote mutations, ARC syndrome, VPS33B

## Abstract

**Background:**

Arthrogryposis-renal dysfunction-cholestasis (ARC) syndrome is a rare disease with a high mortality rate caused by VPS33B or VIPAS39 mutations. ARC syndrome typically presents with arthrogryposis, renal tubular leak and neonatal cholestatic jaundice, and most patients with this disease do not survive beyond one year.

**Case presentation:**

Here, we report the case of a 13-year-old girl with ARC featuring an incomplete and mild phenotype with novel compound heterozygous mutations of VPS33B. The patient presented with arthrogryposis (claw-shaped limbs), ichthyosis, jaundice, and pruritus. Laboratory tests revealed highly evaluated levels of total bilirubin (TB), direct bilirubin (DB), and total bile acid (TBA) as well as normal levels of gamma-glutamyltransferase (GGT). However, signs of renal dysfunction, as well as other manifestations of ARC syndrome, including nervous system abnormalities, deafness, and failure to thrive, were not observed. The patient’s clinical symptoms of jaundice and pruritus were significantly alleviated by administration of ursodeoxycholic acid. Whole-exome sequencing (WES) revealed novel compound heterozygous mutations of VPS33B, c.1081 C > T (p.Q361X,257)/c.244 T > C (p.C82R). Both variants were predicted to be pathogenic in silico and have never been reported previously. To date, the patients’ cholestatic jaundice has been well controlled with continuous treatment of ursodeoxycholic acid.

**Conclusions:**

We report the case of a Chinese female with ARC including novel compound heterozygous mutations of *VPS33B* and an incomplete and mild phenotype. Early diagnosis and suitable symptomatic therapies are critical for the management of ARC patients with mild manifestations and prolonged lifespan.

## Background

Arthrogryposis-renal dysfunction-cholestasis (ARC) syndrome is an autosomal recessive disorder caused by VPS33B or VIPAS39 mutations. VPS33B is a protein coding gene for vacuolar protein sorting 33 homologue B, which is expressed widely in tissues across the body and plays a critical role in maintaining cell polarity [1]. This syndrome involves multiple tissues, organs, or systems, such as muscle, liver, and kidney [2]. ARC syndrome is an uncommon disease that has a poor prognosis. Most of the reported patients presented some characteristic clinical features, including hypotonia with arthrogryposis, renal dysfunction, failure to thrive, neonatal cholestasis featuring low gamma-glutamyl transferase (GGT), and a high risk of haemorrhage caused by platelet dysfunction [2, 3]. Ichthyosis, cardiovascular disease, deafness, and other additional manifestations are also usually found among ARC syndrome patients. Studies have shown that more than 50% of patients experience dermatologic changes [4]. Few patients with ARC survive beyond one year; most patients die soon after the disease onset [5, 6]. There is a spectrum of phenotypes caused by VPS33B mutations. Patients with the c.1225 + 5G > C mutation in the VPS33B gene may have a mild phenotype and a longer lifespan [7]. Some patients do not present with all manifestations [6, 8], and there are some cases that include high GGT [9]. There is currently no cure for this disease, and further studies on ARC syndrome are urgently needed. Here, we report the case of a 13-year-old Chinese female with ARC syndrome with novel compound heterozygous mutations of VPS33B.

## Case presentation

A 13-year-old girl was admitted to our hospital due to a history of intractable jaundice and skin itching for 1 month. The girl was the first child of a nonconsanguineous couple with unremarkable family history and was born healthy via normal delivery with a birth weight of 3200 g. No obvious or specific abnormalities were found in the girl during the perinatal period. Skin hyperkeratosis and mild limb bone abnormalities were identified during infancy and progressed to ichthyosis and obvious claw-like hands and feet at the age of 6 years. Furthermore, the patient presented with progressive itching and recurrent hand and foot desquamation since 6 years old.

**Fig. 1:**
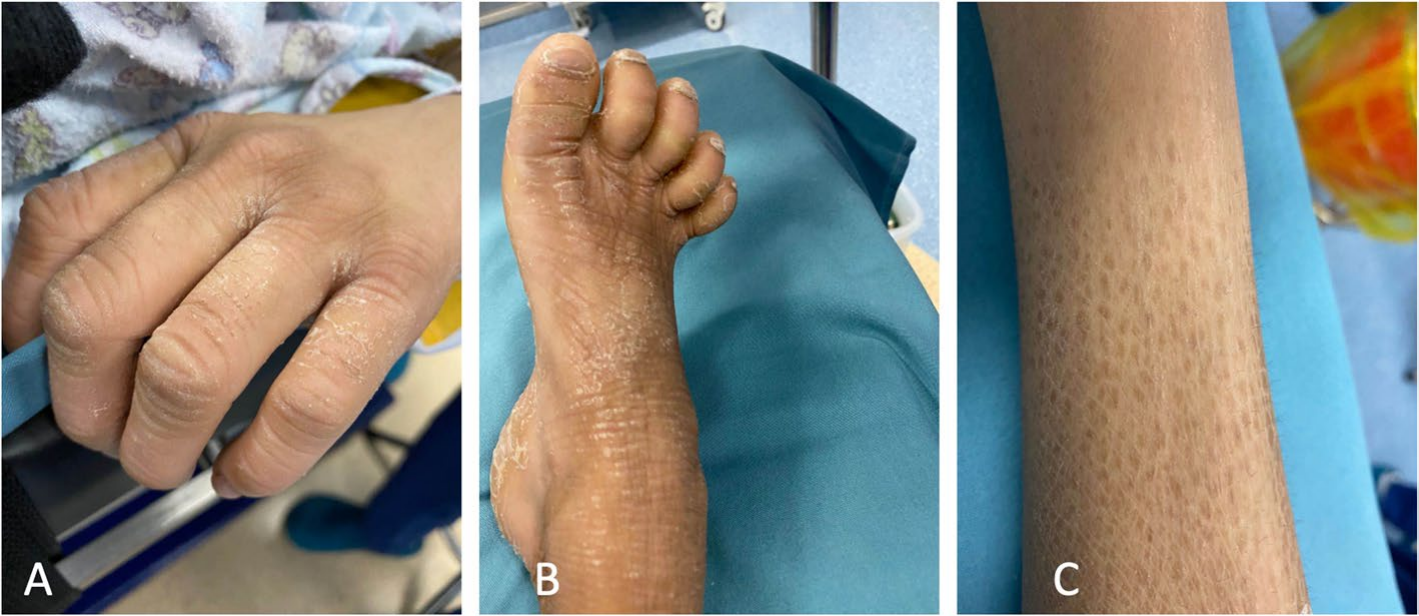
Physical examination of the patient. **A** and **B **Deformities of finger and toe joints (claw-shaped). **C **Fish scale-like skin

**Fig. 2:**
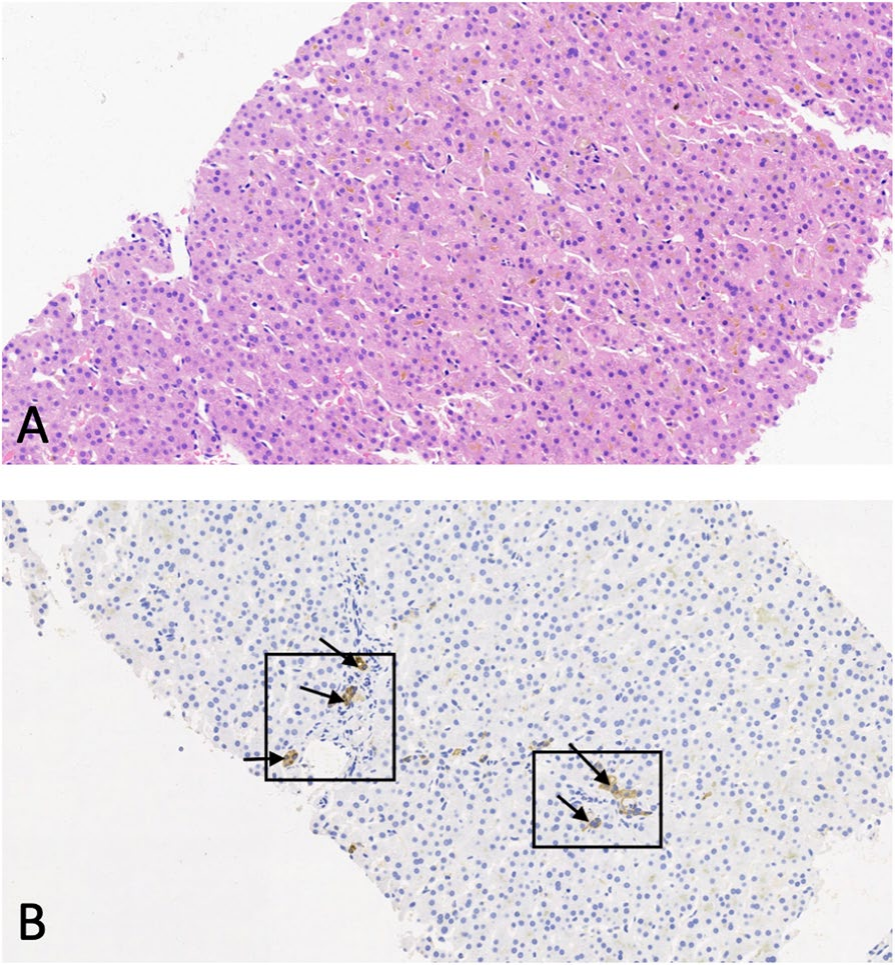
Liver biopsy. **A **Haematoxylin–eosin (HE) staining (200X); **B **Cytokeratin 19 (CK19)-labelled tissues showed the absence of a bile duct (black arrow)

**Fig. 3:**
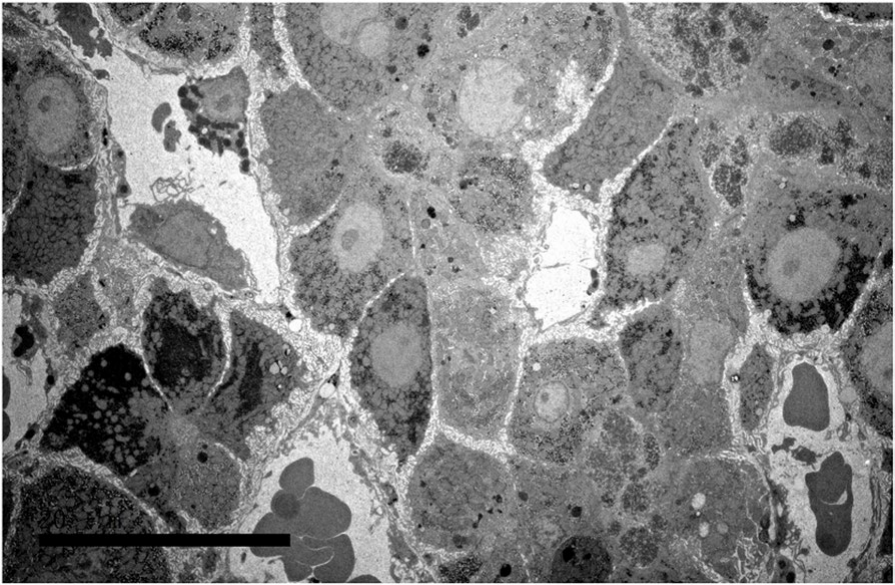
Electron microscopy of the liver tissue showed that the gross bile ducts were severely dilated

One month before admission to our hospital, she was referred to a local hospital for medical support because of worsening jaundice and severe skin itching. The patient was diagnosed with acute liver failure, cholestasis, hypoalbuminemia, and abnormal blood coagulation in the local hospital and was managed with a series of treatments, including hepatoprotective treatment, plasma exchange, albumin infusion, and methylprednisolone pulse therapy. The patient’s liver function, hypoalbuminemia and blood coagulation function improved, while jaundice and skin itching were persistent. The patient was suspected to have a genetic disorder and was recommended for genetic testing. Then, she was discharged from the local hospital after drawing peripheral blood for whole-exome sequencing (WES). On admission, physical examination revealed normal growth with a height of 150 cm and a weight of 38 kg. Moderate skin yellowing, arthrogryposis (claw-shaped limbs), (Fig. 1 A, B), ichthyosis (Fig. 1C), and mild hepatomegaly were observed. The patient presented with mild gait abnormality and walked unsteadily on a broad base. Her muscle tone and strength were normal, and her neurological examination was normal. In addition, her hearing and vision screenings were normal. Laboratory tests showed normal routine blood testing results. Liver function tests revealed increased levels of alanine aminotransferase (ALT, 59 U/L, reference range: 5–40 U/L), direct bilirubin (DB, 117.3 μmol/L, reference range: 0–6.8 μmol/L), (TB, 231.92 μmol/L, reference range: 3.4–17.1 μmol/L), and total bile acid (TBA, 210.5 μmol/L, reference range: 0–10 μmol/L), while aspartate aminotransferases (AST) and gamma-glutamyltransferase (GGT) were normal. Multiple urinalysis showed positive urine bilirubin and urobilinogen but without proteinuria and amino aciduria. Her blood coagulation functions were normal. Alpha foetal protein (AFP) was normal, autoantibody analyses were normal, and liver damaging pathogens Epstein Barr virus (EBV), TORCH, hepatitis A, B, C, and E were all normal. Liver biopsy revealed the absence of the bile duct, and the gross bile ducts were severely dilated and filled with fine particles of bile (Fig. 2). Electron microscopy showed hepatocytes of unequal size and disorganized arrangement with dilated hepatic sinuses (Fig. 3). The mitochondria were swollen, the rough endoplasmic reticulum was hyperplastic and vesicular, the lysosomes were increased, and no viral particles were seen (Fig. 3). A previous head MRI from a local hospital was normal. In addition, WGS results revealed novel compound heterozygous mutations of VPS33B, c.1081 C > T (p.Q361X,257)/c.244 T > C (p. C82R), which was inherited from her father and mother as the parents are heterozygote carriers. Neither variant has been reported previously. In silico analysis predicted that the variant c.1081 C > T (p. Q361X, 257) is pathogenic through abolishment of protein translation, and c.244 T > C (p. C82R) is likely pathogenic according to MutationTastor [10]. Finally, the patient was diagnosed with ARC syndrome based on the clinical manifestations and genetic testing results.

During hospitalization, the patient was treated with ursodeoxycholic acid (250 mg, TID) for cholestasis, methylprednisolone sodium succinate (10 mg, QD) for inflammation, and cholestyramine (4 g, QD) for pruritus. External applications of triamcinolone acetonide econazole cream and urea ointment were used to relieve ichthyosis. Fortunately, the patient’s cholestasis and liver function improved after 1 week of treatment. Her symptoms of jaundice and skin itching were relieved significantly; however, the dermal and arthrosis manifestations persisted. The patient was discharged after 10 days of hospitalization and was managed with maintenance treatment of ursodeoxycholic acid (250 mg, TID). To date, cholestatic jaundice has been well controlled, and her daily quality of life has improved (Table 1).

**Table 1:** Main results of patient’s liver function during follow-up

Biochemical indices	Reference	20.09.10	20.9.16	20.10.22	21.7.28	21.7.31
TB (μmol/L)	3.4–17.1	231.92	156.72	32.74	16.18	10.45
DB (μmol/L)	0–6.8	117.3	77.5	12.00	2.40	1.80
ALT (U/L)	5–40	59	90	76	24	20
AST (U/L)	8–40	39	66	71	31	27
GGT (U/L)	7–32	23	21	15	18	17

*TB* Total serum bilirubin, *DB* Direct bilirubin, *ALT* Alanine aminotransferase, *AST* Aspartate aminotransferase, *GGT* Gamma-glutamyltransferase

## Discussion and conclusions

Generally, most ARC cases are often diagnosed in infancy, as the onset of this disease is usually shortly after birth, and it has a rapid progression. The major causes of death include recurrent infection, dehydration or bleeding [11]. Our patient presented only 3 typical symptoms of cholestasis, arthrogryposis, and ichthyosis. Compared to typical ARC syndrome patients, the patient’s renal function is normal based on urine test results. No renal tubular dysfunction, such as renal tubular acidosis, nephrogenic diabetes insipidus, or glucosuria, appeared. She went for clinical advice because of jaundice and itchy skin, but when we examined her medical history, she showed a characteristic presentation of this disease at an early age. After birth, her skin gradually showed an appearance similar to fish scales with thickened stratum corneum, and her limbs gradually became deformed like claws. At the age of 6 years old, the patient’s dermal manifestations became more similar to the characteristic appearance of ichthyosis with skin itching, which worsened with her growth. Together with her clinical symptoms, signs and genetic finding of pathogenic VPS33B mutations, the patient was diagnosed with ARC syndrome at the age of 13 years old. The delayed diagnosis of our case may be due to early ARC symptoms (icthyosis and arthrogryposis) not being recognized as part of the ARC syndrome, possibly due to a lack of jaundice, and it may be that many physicians overlooked the diagnosis due to its rarity and lack of expertise. Although most of the reported ARC patients died in their early ages (< 12 months), patients with incomplete and mild phenotypes are likely to achieve a prolonged lifespan. In addition to the patient presented in this study, several patients with extended lifetimes who had similar conditions of mild symptoms and slow progression were reported [8]. Five reported ARC syndrome patients with attenuated or incomplete phenotypes are summarized in Table 2. All these patients had a relatively long lifespan, although their growth failure and quality of life could not be improved significantly [6, 12, 13]. The prolonged lifespan of ARC patients may be attributed to mild symptoms and could be managed by symptomatic treatments. Currently, our patient is 14 years old, and her cholestasis was alleviated by maintenance treatment with ursodeoxycholic acid. Her daily quality of life is improved, and she is expected to have a longer life despite the persistent dermal manifestations and arthrosis symptoms.

**Table 2:** Brief summarization of reported attenuated or incomplete cases of ARC syndrome

Reference	Age	Mutation	Typical Phenotype	Other manifestations	Examinations	Treatments	Prognosis
[13]	2.5y	c.240–577_290-156del c.1225 + 5G > C	ⒶⓇⒸ	1. Ichthyosis 2. Pruritus 3. Growth failure 4. Hearing loss	MRI: dysmorphic ventricle	1. Conventional treatments 2. Cutaneous biliary diversion 3. Supplemental feeds via gastric tube	1. Cholestasis and ichthyosis showed no response to conventional therapy 2. Ichthyosis improved after cutaneous biliary diversion 3. Growth improved with supplemental feeds via gastric tube
[13]	12 m	c.1261_1262delCA c.1225 + 5G > C	ⒶⓇⒸ	1. Pruritus 2. Growth failure 3. Hearing loss 4. Hypercholanemia 5. Abnormal dental composition	MRI: hin corpus callosum and diffuse paucity of white matter	1. Rifampicin, phenobarbitone, and ursodeoxycholic acid treatment 2. Hearing aid 3. Dysplasia corrective surgery	1. Language skill improved with hearing aid 2. Growth failure and pruritus wasn’t improved
[7]	7.7y	c.1157A > C (p.His386Pro)	ⒶⓇⒸ	1. Growth failure 2. Dry skin	MRI: marked hypoplasia of corpus callosum, decrease in white matter volume, increased T1 signal in basal ganglia	Biliary diversion	Pruritus improved
[7]	3y	c.1157A > C (p.His386Pro)	ⒶⓇⒸ	1. Growth failure 2. Ichthyosis	MRI: thin and hypoplastic corpus callosum, white matter hypoplasia and delayed myelination, increased signal in basal ganglia	Biliary diversion	Pruritus improved
[7]	11y	1225 + 5G > C and partial deletion in the VPS33B gene	ⒶⓇ	1. Ichthyosis 2. Hearing loss 3. Growth failure	Ultrasound: small kidneys; echogenic liver without hepatosplenomegaly	Potassium citrate and enalapril	N/A

The mechanisms of ARC syndrome are not completely understood, but it is believed to be associated with abnormal expression of VPS33B protein caused by VPS33B mutations. The VPS33B protein is a Sec1/Munc18 family protein involved in membrane trafficking through interacting with the Rab11 family at recycling endosomes that plays a key role in establishing structural and functional aspects of hepatocyte polarity [14]. The deficiency of VPS33B protein leads to mislocalization of canalicular proteins, such as ATP binding cassette subfamily B member 11 (ABCB11), in hepatocytes that influence biliary physiology [14, 15]. In healthy individuals, bile acid is secreted via ABCB11 in hepatocytes. VPS33B protein combines with apical-basolateral polarity regulator (VIPAR), which forms a complex that helps the trafficking of ABCB11 towards the canalicular side of hepatocytes by interacting with apical recycling endosomes (AREs). Mutations in *VPS33B* lead to a malfunctional VPS33B/VIPAR complex, which cannot bind with ARE, causing ABCB11 to move to the basolateral side and leading to cholestasis in ARC patients [14, 15]. Furthermore, hepatic VPS33B deficiency was demonstrated to aggravate cholic acid-induced cholestatic liver injury in mice [16]. Ichthyosis presented in ARC patients is also a result of insufficiency of VPS33B, as the lack of VPS33B protein can lead to unusual epidermal lamellar bodies and abnormal stratum corneum formation construction [17]. *VPS33B* knockout mice showed a similar skin appearance to ARC syndrome patients [18]. In addition to ARC, *VPS33B* mutations can cause another similar disease, autosomal recessive keratoderma-ichthyosis-deafness syndrome (ARKID) [19]. The typical manifestations of ARKID include ichthyosis, hearing loss, severe failure to thrive and osteopenia [19, 20]. Normally, doctors can differentiate ARKID and ARC in patients with *VPS33B* mutations according to the typical symptoms. Nevertheless, further research is needed to investigate the correlations between *VPS33B* mutations and clinical manifestations in both ARKID and ARC patients.

Currently, there is no cure for ARC syndrome. Symptomatic therapies are mostly used to manage patients with ARC syndrome. Fortunately, the major manifestation of our patient, i.e., cholestasis, was significantly relieved by ursodeoxycholic acid during hospitalization and is well-controlled by maintenance treatment of ursodeoxycholic acid. In most cases, conventional treatments are always useless, while biliary diversion can relieve pruritus in some mild cases [6, 12, 13]. In addition, some methods, such as low-density lipoprotein apheresis and biliary diversion, have been reported to improve symptoms, especially pruritus, but the efficacy for improving prognosis was not satisfactory [21]. A report showed that liver transplantation may provide a promising way to cure cholestasis in patients [22]. Thus, genetic counselling before pregnancy and prenatal genetic testing are important for families with a history of ARC syndrome due to its poor prognosis [23].

In summary, our presented case features a prolonged survival time of ARC syndrome. Together with other reported cases with prolonged lifespan, the prognosis of ARC patients likely depends on the gene mutation type, phenotype and time of disease onset with unrevealed mechanisms. Thus, early diagnosis and suitable supporting treatments are important in improving the quality of life of patients with ARC syndrome.

## Data Availability

The original contributions presented in the study are included in the article or supplementary materials, further inquiries can be directed to the corresponding author.

## Abbreviations

ARC syndrome: Arthrogryposis-renal dysfunction-cholestasis syndrome
ALT: Alanine aminotransferase
AST: Aspartate aminotransferases
TBA: Total bile acid
GGT: Gamma-glutamyltransferase
WES: Whole-exome sequencing
DB: Direct bilirubin
TB: Total bilirubin
ARKID: Keratoderma-ichthyosis-deafness syndrome
